# A Near Zero Refractive Index Metamaterial for Electromagnetic Invisibility Cloaking Operation

**DOI:** 10.3390/ma8084790

**Published:** 2015-07-29

**Authors:** Sikder Sunbeam Islam, Mohammad Rashed Iqbal Faruque, Mohammad Tariqul Islam

**Affiliations:** 1 Centre for Space Science, Research Centre Building, Universiti Kebangsaan Malaysia, Bangi 43600, Malaysia; E-Mail: rashed@ukm.edu.my; 2 Department of Electrical, Electronic and Systems Engineering, Faculty of Engineering and Built Environment, Universiti Kebangsaan Malaysia, Bangi 43600, Malaysia; E-Mail: tariqul@ukm.edu.my

**Keywords:** cloak, metamaterial, near zero refractive index (NZRI)

## Abstract

The paper reveals the design of a unit cell of a metamaterial that shows more than 2 GHz wideband near zero refractive index (NZRI) property in the C-band region of microwave spectra. The two arms of the unit cell were splitted in such a way that forms a near-pi-shape structure on epoxy resin fiber (FR-4) substrate material. The reflection and transmission characteristics of the unit cell were achieved by utilizing finite integration technique based simulation software. Measured results were presented, which complied well with simulated results. The unit cell was then applied to build a single layer rectangular-shaped cloak that operates in the C-band region where a metal cylinder was perfectly hidden electromagnetically by reducing the scattering width below zero. Moreover, the unit cell shows NZRI property there. The experimental result for the cloak operation was presented in terms of S-parameters as well. In addition, the same metamaterial shell was also adopted for designing an eye-shaped and triangular-shaped cloak structure to cloak the same object, and cloaking operation is achieved in the C-band, as well with slightly better cloaking performance. The novel design, NZRI property, and single layer C-band cloaking operation has made the design a promising one in the electromagnetic paradigm.

## 1. Introduction

Hiding an object or making something invisible, has received a great deal of interest in the scientific community from the beginning of the modern age. For this purpose, various approaches were imagined but none of them were as notable an achievement as the advent of metamaterials. Metamaterials are a kind of composite material that may exhibit some extra ordinary electromagnetic properties. With the advent of metamaterials, the fantasy of invisibility has gone from fiction to reality. After the first successful demonstration of metamaterial, exposed in [1], various types of design and applications were proposed in the literature [2,3,4]. Nowadays, utilization of a unit cell has received a great deal of attention by researchers, instead of bulk metamaterials, especially in the fields of antenna design, filter design, *etc*. [5,6]. In addition to these fields, metamaterials are widely being used in many other important applications, for instance SAR (specific absorption rate) reduction [7], absorber design [8], polarizer design [9], invisibility cloak design [10], and in other applications [11]. In the field of electromagnetic cloaking operations, metamaterials have played a very significant role. An electromagnetic cloak is a device where an object is made invisible. An object can be made invisible if it does not redirect electromagnetic waves back to the source, or it does not scatter electromagnetic waves in any directions, which means that the object should not disturb the field beyond itself. Cloaking is usually important for military applications, stealth coating of aircraft or missile, reducing the communication interference in the urban area, *etc*. [12]. It is known that electromagnetic waves contain both electric and magnetic fields. An ordinary optical material usually only affects the electric fields, whereas metamaterials can interact with the magnetic components of an electromagnetic wave as well. In addition to other interesting properties of metamaterial, the “Near zero refractive index (NZRI)” property has good potential for designing such a cloak [13]. Several strategies were applied in the literature for performing cloak operations, such as transformation optics, scattering cancellation technique, geometric optics, topology optimization, *etc*. After the realization of the first cloak design, based on transformation optics [10], many such works were performed [14,15,16]. In transformation optics (TO), a volume of space is created where virtually no field exists, but, instead, electromagnetic waves are actually guided around the volume, which effectively makes the volume electromagnetically undetectable. One of the advantages of the TO technique is that it is independent of the shape of object being cloaked, and Maxwell’s equations are form-invariant to also coordinate transformations. The “Scattering cancellation” technique is another familiar method to cloak an object with a metamaterial shell, where scattering from the object being cloaked is reduced to zero [17,18]. For that purpose, scattering cross-section (SCS) is used as a metric to judge the performance of the cloak. A good cloaking device maintains an SCS below one. Usually, epsilon negative metamaterial or materials having less than zero effective permittivity can be utilized as a cloak shell to reduce the SCS. However, the reduction of SCS has been also realized for transformation optical design [19]. In recent works, the utilization of a metasurface instead of bulk metamaterials has received a great deal of attention by researchers as an alternative approach to reduce the SCS [20,21,22,23,24,25,26]. This type of surface offers similar exotic properties to those of metamaterials, but in easier way and with better functionality. A cloaking operation that is performed using a metasurface is called a “*Mantle Cloak*”. The advantages of a *mantle cloak* may include simplicity, design flexibility, conformability, better bandwidth capability, *etc*. Topology optimization is a new approach that makes the optimization of a cloak layout easy [27]. However, this approach was only investigated for cylindrical cloaks. There are some other cylindrical cloaks are found in the literature, such as Alitalo *et al.* [28] who claimed a single layer cylindrical cloak that works in the S-band (3.3 GHz). Shin *et al.* [29] demonstrated a metamaterial-based ground plane cloak, but it only works in the X-band. Matekovits *et al.* [30] developed and utilized a single layer material of width modulated unit cells, but it only works in the K-band.

In this paper, a metamaterial unit cell is proposed that exhibits near zero-refractive index property in the C-band. The proposed material was utilized as a shell to build a single layer rectangular cloak, and it also operates in the C-band. Some topology optimization based studies were performed, based on the shape (triangular and diamond shape) of the cloak, to optimize cloak operation in the C-band.

## 2. Materials and Method

**Figure 1:**
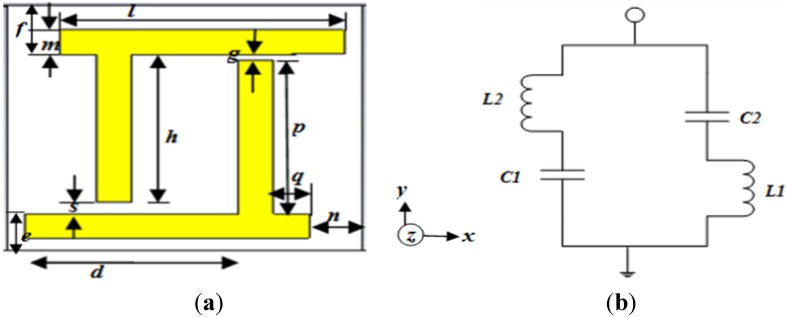
(**a**) The proposed unit cell structure; (**b**) equivalent circuit of the unit cell.

The design parameters of the unit cell of the proposed material are shown in Figure 1a. In the basic design, two orthogonal metal arms of copper were conversely placed in such a way that it forms a near-pi-shape structure. To form a near zero refractive index property, the series branch of the unit cell circuit must contain an inductance or capacitance, and the corresponding shunt branch must contain the opposite order of reactance. Therefore, two unequal gaps were maintained at each end of the arms to form the capacitive effect, denoted by “*g*” and “*s*”. Conversely, each unequal metal arm is responsible for generating inductance. The thickness of all the copper lines were kept at 0.035 mm. The equivalent circuit is presented in Figure 1b. Due to the unequal gap and length of each metal link, the inductance and capacitance were named in the figure as *L1*, *L2* and *C1*, *C2*, respectively. The whole structure was designed over a FR-4 substrate material that has a dielectric constant of 4.2 and dielectric loss-tangent of 0.002. The length and width of the substrate was kept at 10 mm and the thickness of the substrate was maintained at 1.6 mm. The design specifications are seen in Table 1.

**Figure 2:**
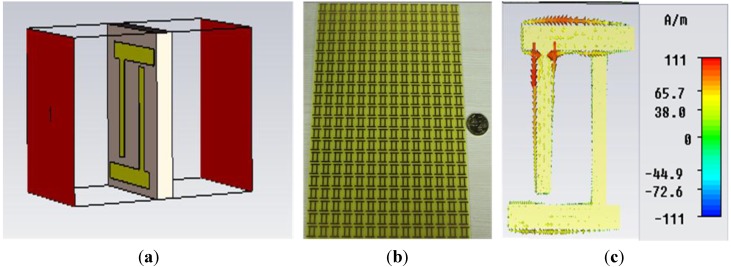
(**a**) Simulation geometry for *z-axis* wave propagation; (**b**) fabricated prototype for measurement; (**c**) current distribution of the unit cell at a frequency of 8.29 GHz.

For the numerical analysis of the unit cell, the finite integration technique based CST Microwave Studio simulation software was utilized. The two wave-guide ports were placed at the positive and negative end, on the *z-axis* of the unit cell structure, and the transverse electromagnetic wave was propagated between the ports. The simulation geometry is shown in Figure 2a.

**Table 1:** Unit cell parameters.

Unit cell parameters	Value (mm)
*d*	6
*e*	1.5
*f*	2
*g*	0.33
*h*	6
*l*	8
*m*	1
*n*	1.5
*p*	6.27
*q*	1
*s*	0.5

For measurement purposes, a prototype of 140 × 230 mm^2^ was fabricated, which was composed of 14 × 23 unit cells, as seen in Figure 2b. The two horn antenna were placed at the two sides of the prototype in an open space environment, in such a way so that *z-axis* wave propagation was ensured. The antennas were connected to a vector network analyzer N5227A, which had been used for the calculation the the S-parameters of the unit cell. Moreover, for calibration purposes, measurements with and without prototype were performed as well.

## 3. Results and Discussion

The current distribution of the unit cell at a frequency of 8.29 GHz is shown in Figure 2c. Unlike the split ring resonator, the current is following the opposite direction in the two arms of the unit cell because of the dissimilar geometry of the unit cell.

**Figure 3:**
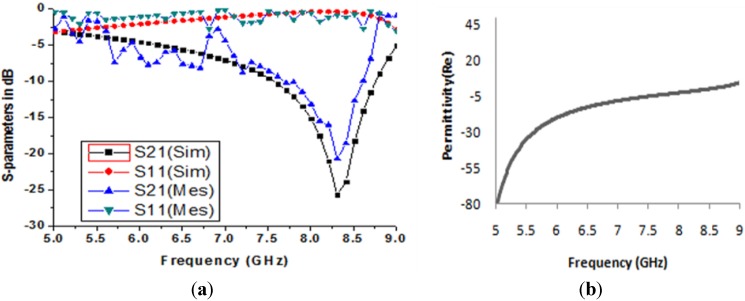
(**a**) Numerical and measured transmission (S_21_) and reflection coefficient (S_11_) for the unit cell structure; (**b**) Real value of effective permittivity *versus* frequency for *z-axis* wave propagation.

In Figure 3a, both the numerical and experimental magnitudes of the S-parameters are shown. The figure shows one broad resonance at a frequency of 8.29 GHz of the transmission coefficient (S_21_). The measured result is presented in the same figure, which almost agrees well with the simulated results. However, a slightly shifted and shortened transmittance is found at the frequency of 8.37 GHz when compared to the simulated results. This shift usually occurs due to an open space measurement process or fabrication errors.

**Figure 4:**
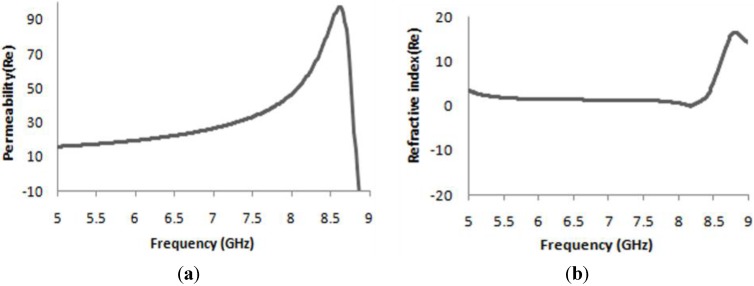
(**a**) Real value of effective permeability versus frequency; (**b**) Real value of refractive index (η) *versus* frequency for *z-axis* wave propagation.

The effective medium parameters, the permittivity (*ε*), permeability (*μ*), and refractive index (*η*) were calculated from the S-parameters using the method mentioned in [31]. In Figure 3b, the real magnitude of effective permittivity (*ε*) is seen. At a frequency of 8.29 GHz, the real value of the permittivity is negative, with *ε* = −1.15. In Figure 4a, the real value of the effective permeability is shown. At a frequency of 8.29 GHz, the real magnitude of the effective permeability shows a clear positive magnitude. After a frequency of 9 GHz, the permeability curves become negative. This usually happens at a higher frequency, because, at this frequency, the current of the oscillator cannot remain in phase with the applied field.

Similarly, Figure 4b reveals the real peak of refractive index where more than 2 GHz (from the frequency of 5.20 GHz to 8.48 GHz) wideband near zero refractive index (NZRI) property is being displayed. The NZRI property is a very good prospect in the field of cloaking and directional antenna design. Moreover, at a frequency of 8.29 GHz, the real magnitude of refractive index shows a positive peak with a value of *η* = 0.89 for *z-axis* wave propagation.

### 3.1. Design and Analysis of A Rectangular Cloak Using The Metamaterial

**Figure 5:**
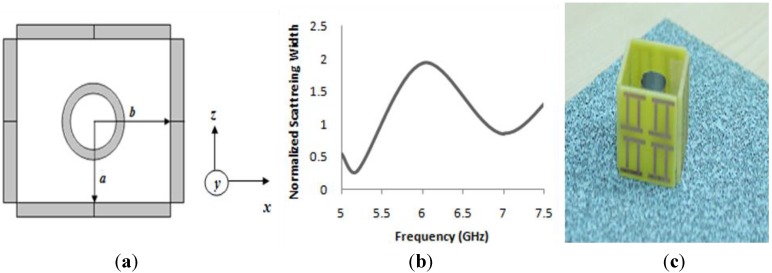
(**a**) Geometry of the rectangular cloak with metal cylinder (inside); (**b**) Numerical result of normalized scattering width of cloaked object normalized to scattering width of bare object; (**c**) Cloak structure for measurement.

As a part of further investigation, the proposed material was initially utilized to design a rectangular cloak. In this study, the scattering cancellation technique was adopted to convince the cloaking operation. As we stated earlier, in the scattering cancellation technique, the object to be cloaked is surrounded by a kind of dielectric shell that will cancel scattering from the core and it will return the electromagnetic waves in their original path. For this reason, in this study, four 20 × 20 mm^2^ walls, composed of the proposed material, were utilized as a shell, where each wall contains 2 × 2 proposed unit cells. An aluminium cylinder was placed inside the cloak shell in such a way that the distance from the centre of the cylinder to each of the surface wall of the cloak was a = b = 10 mm. The height of the cylinder was 20 mm; the inner and outer radii of the cylinder were 3 mm and 4 mm, respectively. The object with a cloak shell is presented in Figure 5a. To show the cloaking effect, the performance of the cloaked object, as well as the performance of the bare object, are usually compared. Therefore, for numerical analysis, the cloak shell with the metal cylinder inside of it was illuminated by a plain wave that was propagating through the *x*-axis with an electric field along the *y-axis*. A similar methodology was applied for the bare cylinder as well. To evaluate the cloaking effect qualitatively, the total scattering width is calculated, which is defined by the total energy scattered from the object, normalized to the incident energy. For that calculation, the equation that was mentioned in [32] has been utilized.

In Figure 5b, the numerical result are shown for the total scattering width as a function of frequency for the cloaked object normalized by the total scattering width of the bare object that was calculated in the *xy-plane*. It is evident from Figure 5b that, at the frequency of 5.18 GHz, the calculated normalized scattering width curve was reached at a value lower than zero with a value of 0.10, which indicates that the object has been cloaked perfectly at that frequency. In fact, the normalized scattering width graph has started showing a value lower than zero from the frequency of 5 GHz to 5.35 GHz in the C-band of microwave frequency regime. Therefore, this frequency range can be also considered as a cloaked frequency zone.

Figure 5c shows the cloak structure prepared for measurement purposes with the metal cylinder inside. Similar to the arrangement for the simulation, a cloak structure was prepared for measurement purposes. For the measurement of the cloaking performance, two WR137 C-band rectangular waveguides were utilized. Moreover, a copper box with 44 mm long, 68.5 mm wide, and 49.4 mm height was prepared, according to the size of the waveguide, which was open at its two ends. The copper box was slightly bigger than each waveguide so that two waveguides can be inserted into the box, keeping them face-to-face. This box was placed between two waveguides and the cloak structure was placed inside the box for measurement. For measurement purposes, a metal cylinder, with cloak structure and without cloak structure, was measured. The waveguides were connected to the same vector network analyzer (N5227A) and the S-parameters were calculated.

**Figure 6:**
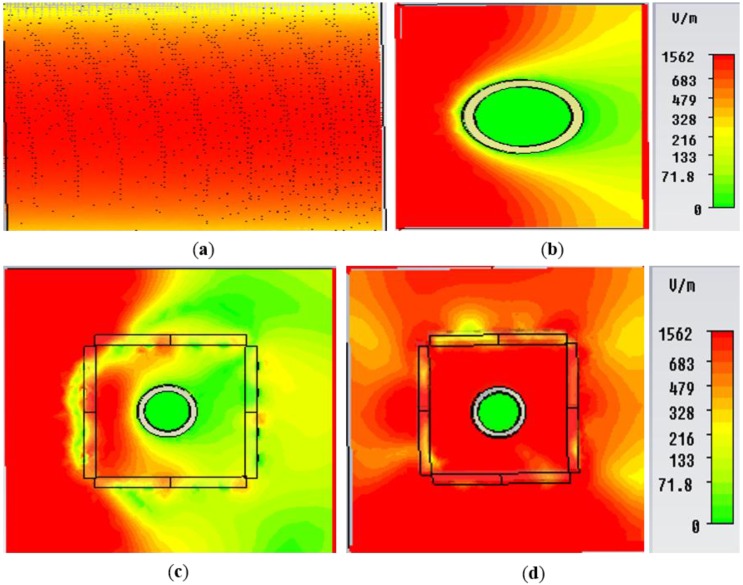
E-field distribution in the *xy-plane* for (**a**) free space; (**b**) bare object; (**c**) object at uncloaked frequency; (**d**) object at cloaked frequency (at 5.18 GHz) obtained from CST Microwave Studio.

**Figure 7:**
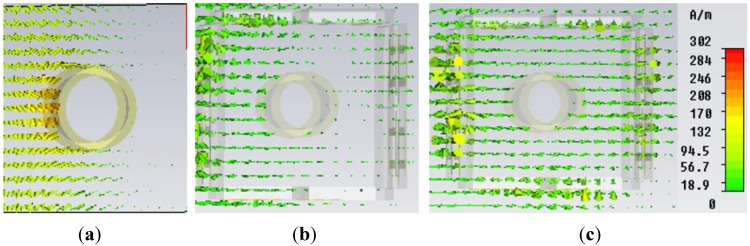
H-field distribution in the *xy*-plane for (**a**) bare object; (**b**) object at uncloaked frequency; (**c**) object at cloaked frequency (at 5.18 GHz) obtained from CST Microwave Studio.

In Figure 6, the electric field distribution in the *xy-plane* for the free space, bare object, objects at uncloaked and at cloaked frequencies are presented. In Figure 6a, the free space field map is shown. Similarly, in Figure 6b, it is apparent from the field map that a strong E-field distortion is clearly visible for the bare target. It indicates shadow in the forward direction (behind the bare target) and reflection in the backward direction. The shadow reveals the scattering in the forward direction. A similar effect is seen for the object at an uncloaked frequency in Figure 6c. However, in Figure 6d, the object at a cloaked frequency (5.18 GHz) is displayed, where fine wave front reconstruction is clearly seen and the shadow behind the cloaked object is effectively alleviated. Here, the reason is that the material is cancelling the scattered waves and dipole moment of opposite sign it is inducing there. Therefore, it brings the plain waves to their original path. In Figure 7, similar effects for the bare object, object at uncloaked and at cloaked frequencies are presented for H-field distribution, which also displays the same behavior of the E-field distribution.

**Figure 8:**
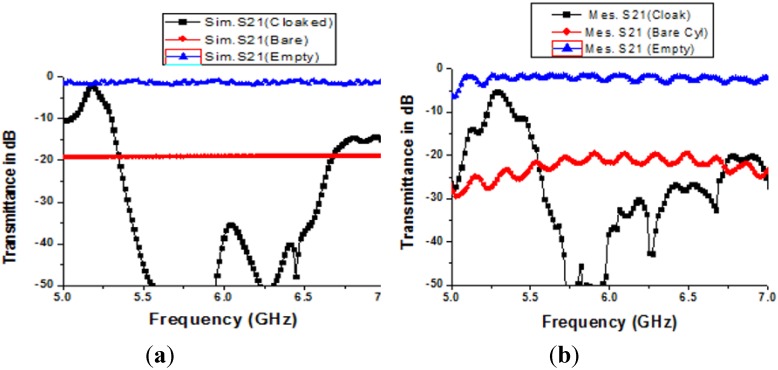
(**a**) Numerical results of the S-parameters for the cloaking operation; (**b**) measured results of the S-parameters for the cloaked state.

In Figure 8a,b, the numerical and measured results of the transmission parameter (S_21_) for both the uncloaked and cloaked states are shown, respectively. In this study, the S-parameters are compared with the empty state. The “empty state” refers to the measured free space transmittance (S_21_) that was taken by the usual free space measurement process using two horn antennas and the network analyzer N5227A, and the free space transmittance was found near the zero line. The “bare” line refers to the characteristics of the bare cylinder only, without the cloak shell and the “cloak” line defines the metal cylinder within the material cloak shell.

In Figure 8a, the numerical results are presented where the transmission coefficient (S_21_) has touched the free space line at a frequency of 5.25 GHz, which is in the C-band and it is also far from the bare cylinder characteristics. Consequently, at this frequency, the S_21_ and empty S_21_ are nearly same and the object has been cloaked properly at this point of frequency. Therefore, if any outsider sees the object at this frequency, he will see the free space line instead of the object characteristics. Moreover, the normalized scattering width at this frequency exhibits a less than zero value as well. Similarly, in Figure 8b, the experimental results are presented for the cloak, where transmission characteristics are found to be nearly same as the numerical results. The experimental transmittance for the object within cloak shell displays a peak very close to zero, at a frequency of 5.29 GHz instead of at the 5.25 GHz of the simulated results. Therefore, the measured results show as slightly right shifted, but with almost good conformity to the simulated results. This shift may occur due to a fabrication error or an inter shell gap error of the materials.

### 3.2. Design and Analysis of An “Eye-Shaped” Cloak Using The Metamaterial

**Figure 9:**
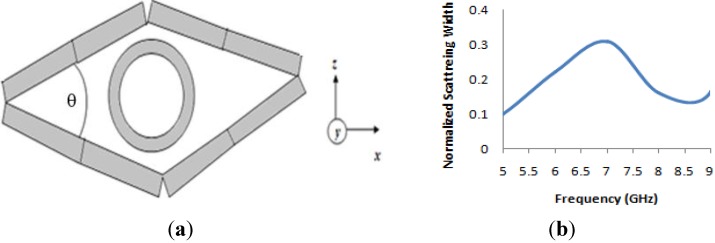
(**a**) Geometry of the “eye-shaped” cloak with metal cylinder (inside); (**b**) numerical result of normalized scattering width of cloaked object normalized to scattering width of bare object for the “eye-shaped” cloak.

In this section, another cloak was designed, based on the same material shell. As a part of topology optimization, an eye-shaped cloaked was designed. The same size and number of material walls were utilized and the four walls were set in such a way that they produced an eye-shaped “cloak”. Figure 9a shows the simulation arrangement of the eye-shaped cloak. Two design an eye-shaped structure, each of the two-walls were connected at one of their ends at an angle of *θ* = 45°, as seen in Figure 9a. For simulations, the same methodology as the rectangular cloak was applied. Figure 9b displays the normalized scattering width of cloaked object normalized to scattering width of bare object for the “eye-shaped” cloak. It is apparent from Figure 9b that the scattering width exhibits a value less than zero at a frequency of 5GHz and 8.70 GHz in the microwave frequency region. This signifies that the object has been cloaked at these (5GHz and 8.70 GHz) frequencies.

**Figure 10:**
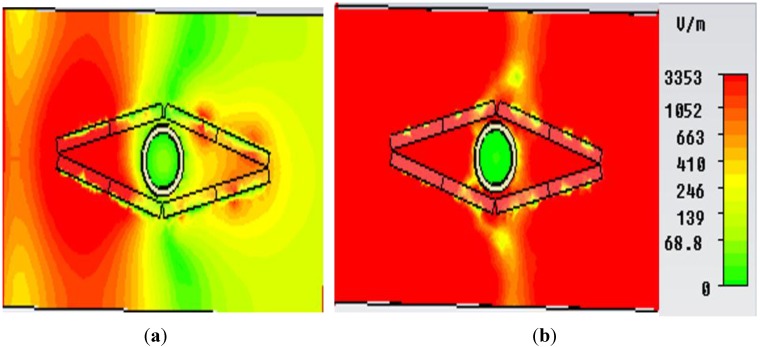
E-field distribution in the *xy*-plane for (**a**) object at uncloaked frequency; (**b**) object at cloaked frequency (at 5 GHz) obtained from CST Microwave Studio for the “eye-shaped” cloak.

**Figure 11:**
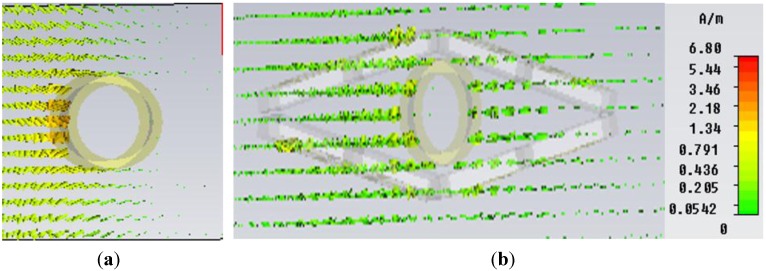
H-field distribution in the *xy-plane* for (**a**) bare object; (**b**) object at cloaked frequency (at 5 GHz) obtained from CST Microwave Studio for the “eye-shaped” cloak.

In Figure 10, the field distribution maps are shown for the object at an uncloaked frequency (Figure 10a) and the object at a cloaked frequency (Figure 10b) for the “eye-shaped” cloak. From Figure 10a, it is clearly visible that, for the object at an uncloaked frequency, scattering in the forward dirrection (*i.e.*, zero field green shadow behind the object) is present. However, according to Figure 10b, in the case of the cloaked frequency (at 5GHz), the shadow behind the object has diminished and fine wave front reconstruction is achieved. In Figure 11a,b the H-field distribution in the *xy-plane* is depicted for the bare object and the object at a cloaked frequency, consecutively, where the H-field ditribution also exhibits similar characteristics of the E-field map.

### 3.3. Design and Analysis of A “Triangular-shaped” Cloak Using The Metamaterial

**Figure 12:**
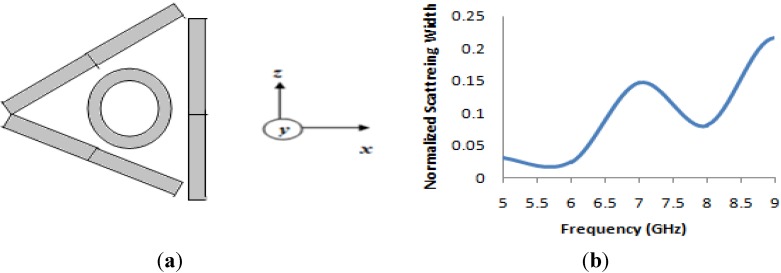
(**a**) Geometry of the “triangular-shaped” cloak with metal cylinder (inside); (**b**) numerical result of normalized scattering width of a cloaked object normalized to the scattering width of the bare object for the “triangular-shaped” cloak.

In this section, a new triangular-shaped cloak is introduced. In this design, three material walls were utilized as a cloak shell. The three equal (like the previous cloak) material walls of the same size were arranged in such a way that they formed a triangular shape. In this design, two material-based walls coincided with each other at a 45° angle and third wall was placed at the back end of these two. The simulation geometry of the triangular-shaped cloak is seen in Figure 12a. For simulation, the same methodology was adopted as with the rectangular cloak using the same object. Figure 12b shows the normalized scattering width of a cloaked object normalized to the scattering width of the bare object for the “triangular-shaped” cloak. It is evident from Figure 12b that the scattering width shows a value less than zero from the frequencies of 5 GHz to 6.5 GHz. Therefore, it signifies that the object is being cloaked in this frequency range. However, the lowest value of the scattering width is achieved at 5.67 GHz with a value 0.024.

**Figure 13:**
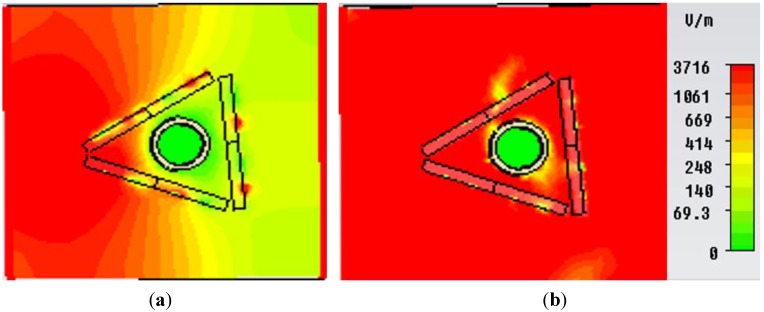
E-field distribution in the *xy*-plane for (**a**) object at an uncloaked frequency; (**b**) object at a cloaked frequency (at 5.67 GHz) obtained from CST Microwave Studio for the “triangular-shaped” cloak.

In Figure 13, the E-field distribution map in the *xy*-plane is shown for the object at an uncloaked frequency (Figure 13a) and object at a cloaked frequency (Figure 13b) for the “triangular-shaped” cloak. In Figure 13a scattering at different direction is seen for object within cloak shell but at an uncloaked frequency. However, it is evident that in Figure 13b, the forward scattering, as well as the scattering in other directions has been reduced for the object within the cloak shell at the cloaked frequency and wave front reconstruction is obtained.

**Figure 14:**
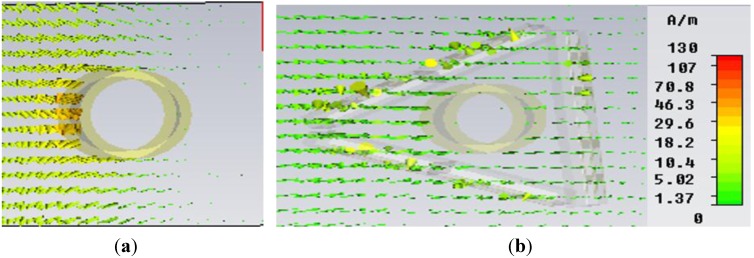
H-field distribution (Hedgehog plot) in the *xy-plane* for (**a**) bare object; (**b**) object at a cloaked frequency (at 5.67 GHz) obtained from CST Microwave Studio for the “triangular-shaped” cloak.

Similarly, Figure 14a and Figure 14b depict the H-field map in the *xy-plane* for the bare object and the object at a cloaked frequency of 5.67 GHz, respectively. It is apparent from Figure 14b that, compared to the field map of the bare object in Figure 14a, no shadow or scattering in the forward direction is visible in Figure 14b for the object at a cloaked frequency in the triangular cloak shell. Therefore, these field maps also reveal the cloaking operation at a frequency of 5.67 GHz in the C-band of microwave frequency regime.

### 3.4. Comparative Analyses of The Three Different Cloak Configurations

In this study, three different types of cloak structure were proposed: rectangular-shaped, eye-shaped, and triangular shaped. In these three configurations, for the rectangular and eye-shaped cloak, the four same size metamaterial-based walls were utilized, whereas for the triangular shape only three walls were utilized.

**Figure 15:**
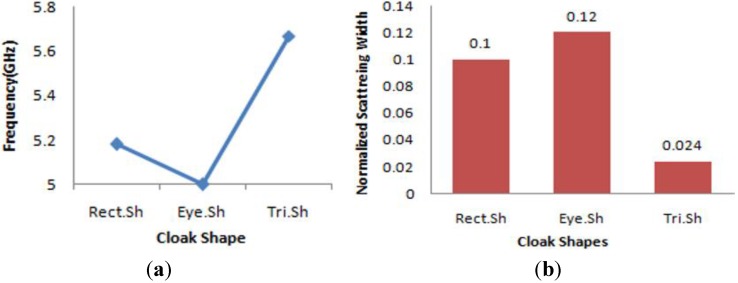
(**a**) Frequency comparisions of the three different cloak shapes; (**b**) NSW comparisions of the three different cloak shapes.

In Figure 15a,b, the comparisions of the three different cloak shapes are given from the frequency and normalized scattering width (NSW) point of view for the same object. From Figure 15a, it is seen that, for both of the rectangular and eye-shape cloak, the lowest NSW was found near 5 GHz, but for triangular-shape cloak the lowest NSW was found at 5.67 GHz. However, all the cloak shapes cloaked the object in the same C-band regime. As a metric of cloaking performance, if the normalized, scattering width is compared among these three shapes for the same object, from Figure 15b; it is evident that the triangular shape has the lowest NSW compared to the others, and the eye-shape cloak has the highest NSW for the object.

## 4. Conclusions

In this paper, a new pi-shaped metamaterial unit cell was introduced that exhibits more than a 2 GHz wideband near zero refractive index (NZRI) property in the microwave region. The material was then utilized in the design of three types of cloak shapes (rectangular-shaped, eye-shaped, and triangular shaped) for hiding a metal cylinder. It was found that, for all the three types of shapes, the metal object could be cloaked in the C-band region by reducing the normalized scattering width to below zero, where the material was showing near zero refractive index property. Therefore, the proposed material and cloak shapes can be utilized, in addition to other metamaterials and cloak shapes, effectively in the C-band microwave spectra.
